# Trial Design and Objectives for Patients With Prostate Cancer: Recommendations From the Prostate Cancer Working Group 4

**DOI:** 10.1200/JCO-25-02834

**Published:** 2026-02-26

**Authors:** Andrew J. Armstrong, Michael J. Morris, Wassim Abida, Rahul R. Aggarwal, Emmanuel S. Antonarakis, Gerhardt Attard, Himisha Beltran, Alan Bryce, Michael A. Carducci, Heather H. Cheng, Delphine L. Chen, Kim N. Chi, Daniel S. Childs, William Dahut, Louise Emmett, Karim Fizazi, Andrei Gafita, Daniel J. George, Ken Hermann, Michael S. Hofman, Thomas Hope, Maha Hussain, W. Kevin Kelly, Elizabeth Kessler, Phillip H. Kuo, Joshua Lang, Glenn Liu, Catherine H. Marshall, Alicia K. Morgans, Rana R. McKay, David Nanus, Peter Nelson, Channing Paller, Zachery R. Reichert, Charles J. Ryan, A. Oliver Sartor, Heiko Schöder, Lawrence H. Schwartz, Nima Sharifi, Walter M. Stadler, Mark Stein, Cora N. Sternberg, Russell Z. Szmulewitz, Scott T. Tagawa, Alexandra O. Sokolova, Alex W. Wyatt, Kosj Yamoah, Evan Y. Yu, Susan Halabi, Howard I. Scher, for the PCWG4 Writing Group, Joshi J. Alumkal

**Affiliations:** ^1^ Department of Medicine, Duke Cancer Institute Center for Prostate and Urologic Cancer, Duke University, Durham, NC; ^2^ Memorial Sloan Kettering Cancer Center, New York, NY; ^3^ San Francisco Helen Diller Family Comprehensive Cancer Center, University of California, San Francisco, CA; ^4^ Masonic Cancer Center, University of Minnesota, Minneapolis, MN; ^5^ University College London Cancer Institute, London, United Kingdom; ^6^ Dana Farber Cancer Institute, Boston, MA; ^7^ City of Hope Cancer Center, Goodyear, AZ; ^8^ Sidney Kimmel Cancer Center, Johns Hopkins University, Baltimore, MD; ^9^ University of Washington and Fred Hutchinson Cancer Center, Seattle, WA; ^10^ BC Cancer Agency, Vancouver, BC, Canada; ^11^ Department of Oncology, Mayo Clinic, Rochester, MN; ^12^ American Cancer Society, Atlanta, GA; ^13^ Department of Theranostics and Nuclear Medicine, St Vincent's Hospital, Sydney, Australia; ^14^ Department of Cancer Medicine, Centre Oscar Lambret, Institut Gustave Roussy, University of Paris Saclay, Villejuif, France; ^15^ Department of Radiology and Radiological Sciences, Johns Hopkins Theranostics Center, Johns Hopkins University, Baltimore, MD; ^16^ Universitätsklinikum Essen, Essen, Germany; ^17^ Peter MacCallum Cancer Centre, Melbourne, VIC, Australia; ^18^ University of California, San Francisco, San Francisco, CA; ^19^ Robert H. Lurie Comprehensive Cancer Center, Northwestern University, Chicago, IL; ^20^ Sidney Kimmel School of Medicine at Thomas Jefferson University, Philadelphia, PA; ^21^ University of Colorado School of Medicine, Aurora, CO; ^22^ Department of Radiology, City of Hope National Medical Center, Duarte, CA; ^23^ University of Wisconsin Carbone Cancer Center, University of Wisconsin, Madison, WI; ^24^ University of California San Diego, San Diego, CA; ^25^ Weill Cornell Medicine, New York, NY; ^26^ Fred Hutchinson Cancer Center, Seattle, WA; ^27^ Department of Medicine, University of Michigan, Ann Arbor, MI; ^28^ Transformational Prostate Cancer Research Center, LCMC Health, New Orleans, LA; ^29^ University of Miami Miller School of Medicine, Miami, FL; ^30^ City of Hope Cancer Center, Chicago, IL; ^31^ Herbert Irving Comprehensive Cancer Center, Columbia University Irving Medical Center, New York, NY; ^32^ Englander Institute for Precision Medicine, Weill Cornell Medicine, New York, NY; ^33^ Department of Medicine, Sandra and Edward Meyer Cancer Center, New York Presbyterian, New York, NY; ^34^ Department of Medicine, University of Chicago, Chicago, IL; ^35^ Division of Hematology and Medical Oncology, Department of Medicine, Weill Cornell Medical College, New York, NY; ^36^ Hematology Oncology, Oregon Health and Science University, Portland, OR; ^37^ Department of Urologic Sciences, Vancouver Prostate Centre, University of British Columbia, Vancouver, BC, Canada; ^38^ Michael Smith Genome Sciences Centre, BC Cancer, Vancouver, BC, Canada; ^39^ Department of Radiation Oncology, H. Lee Moffitt Cancer Center & Research Institute, Tampa, FL; ^40^ James A. Haley Veterans' Hospital, Tampa, FL; ^41^ Department of Biostatistics and Bioinformatics, Duke University Medical Center, Durham, NC; ^42^ University of Melbourne, Parkville, VIC, Australia; ^43^ Weill Cornell Medicine-NewYork Presbyterian Hospital, New York, NY; ^44^ Now at Convergent Therapeutics, New York, NY

## Abstract

**PURPOSE:**

The continuous development of new imaging approaches, molecular phenotyping, genetic subtypes, prognosis assessments, and effective therapies across a range of disease states has created a need to redefine terminology and best practices for clinical trial conduct in patients with advanced prostate cancer.

**METHODS:**

We convened an international expert committee of diverse working groups, the Prostate Cancer Working Group 4 (PCWG4), between 2016 and 2025. Our objective was to formulate updated criteria based on emerging evidence and clinical trial data in a biomarker context to provide guidance for clinical trial design, eligibility, and end point assessments for patients with advanced prostate cancer.

**RESULTS:**

PCWG4 redefines terminology around the disease state and previous therapies in a patient-centric context and terminology focused on androgen pathway modulation. We consider imaging, with a particular focus on positron emission tomography (PET)–defined disease. New recommendations are provided for disease state terminology, defining eligibility criteria, response and delay/prevent end points, intervals for reassessments including imaging, and patient-reported outcome determination. We provide recommendations in a biomarker-based context of use for the intended indication, reflective of patient benefit for specific interventions. We emphasize the need for development of validated PET imaging and molecular and phenotypic criteria as well as trial designs to appropriately risk stratify patients, predict and assess benefit, and measure post-treatment outcomes reliably in a trial framework.

**CONCLUSION:**

PCWG4 updates recommendations on patient and tumor characterization, therapy development, and imaging criteria and extends guidance into earlier androgen pathway modulator–naïve/sensitive disease states to reflect an evolving, heterogeneous, and diverse patient population to optimize treatment benefits for all patients.

## INTRODUCTION

The classification of patients with prostate cancer, based on precision biomarkers and expanding therapeutics, has significantly impacted care and trial conduct over the past decade.^1-8^ With this background, the Prostate Cancer Working Group 4 (PCWG4), an international working group of clinical and translational prostate cancer experts, convened with the goal of standardizing and updating clinical trial guidance.

PCWG3 recommended consideration of all aspects of drug development in a biomarker context.^9^ PCWG4 extends this concept to distinct patient populations with unmet medical needs (Table 1). We account for the complex medical decision making and regulatory environment for an increasingly chronic disease and focus on validated context-specific biomarker assays and devices for contexts of use as defined by the US Food and Drug Administration (FDA) Biomarkers, Endpoints and other Tools (BEST) Resource.^10^ We apply such concepts to patient-centered eligibility and prognostic and predictive biomarkers to select dose and schedules and intermediate end points to detect the emergence of treatment resistance to optimize patient benefits.^11^

**TABLE 1.** Executive Summary of PCWG4 Guidance and Changes

**FIG 1.**
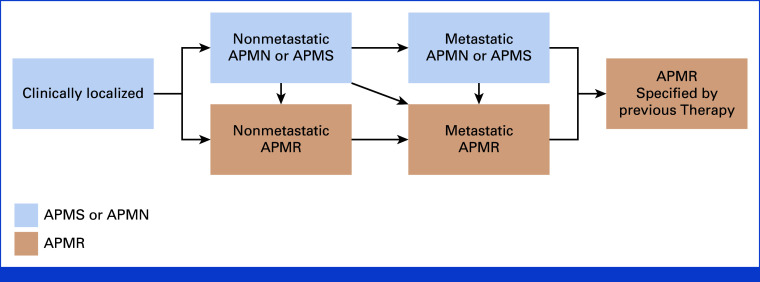
Prostate cancer clinical state model, a framework for patient treatment and drug development, updated for Prostate Cancer Clinical Trials Working Group 4. Combination therapy is considered one line of therapy. Androgen pathway modulation includes ADT and ARPI. Within each state, specify where relevant: (1) genotype (germline, somatic), (2) imaging modality used to define metastasis (PET, CT/MRI/bone scan), (3) disease characteristics and biomarkers critical for risk stratification, including pathology and immunohistochemistry, and (4) previous therapies and outcome including lack of exposure (treatment-naïve), exposed but not resistant, and resistant. APMN/S is the preferred term for hormone-/castration-naïve/castration-sensitive disease (HSPC, CSPC), whereas APMR is the preferred term for castration-/hormone-resistant prostate cancer (CRPC/HRPC). Mapping of previous PCWG3 disease states to the current PCWG4 state model is shown in Appendix Table A1. ADT, androgen deprivation therapy; APMN/S, androgen pathway modulation–naïve/sensitive; APMR, androgen pathway modulator–resistant; ARPI, androgen receptor pathway inhibitor; CSPC, castration-sensitive prostate cancer; CT, computed tomography; HSPC, hormone-sensitive prostate cancer; MRI, magnetic resonance imaging; PCWG, Prostate Cancer Clinical Trials Working Group; PET, positron emission tomography.

List of Changes
Summary of major changes in PCWG4 recommendations compared with the PCWG3 clinical state model
1. Redefines the characterization of patients at a given time point during the disease course, including disease extent, prior therapies, imaging findings, levels of testosterone, genotype/phenotype, ancestry and host factors, and clinical risk (Fig 1, Appendix Table A1) 2. Avoids castration-sensitive and castration-resistant (CSPC/CRPC) nomenclature in favor of a more patient-centric language based on the new therapeutic indication model, including APMN/S and APMR disease, specifying the imaging modality, prior therapies, symptoms, and patterns of spread (Fig 1, Appendix Table A1) 3. Affirms the use of qualified biomarkers (genetic, imaging, pathologic, clinical) to guide precision medicines and therapies to optimize patient benefit and minimize patient risk 4. Emphasizes the importance of serial biological (genetic, phenotypic) profiling of disease at the start of a new therapy and time of progression to redefine emergent actionable alterations and a changing biology. Serial sampling may involve metastatic biopsy, liquid biopsy, and/or tumor-specific imaging
Principles of trial conduct
1. Emphasizes discovering and qualifying post-treatment outcomes that reflect patient benefit or can serve as surrogates of that benefit for use in regulatory submissions to accelerate drug approvals 2. Aligns with FDA BEST guidance for biomarker-defined trial designs 3. Expands criteria/end points to early indications (pre-ADT/ARPI, metastatic and PET only, or nonmetastatic) 4. Highlights the distinction between the need to consistently report measures of progression in a trial *v* the clinical need to continue a particular therapy beyond progression as long as the patient is benefiting from the treatment
Eligibility for enrollment
1. Focuses on increasing enrollment to accurately reflect the demographics of affected patients, particularly those at highest risk of prostate cancer morbidity and mortality 2. Defines eligibility criteria using validated clinical, PET/radiographic, patient-reported, and biological parameters across disease states/indications intended to homogenize the prognosis of the patients enrolled while enriching the prognosis for those most likely to respond to a particular therapy (Tables 2 and 3) 3. Expands pathologic assessments to include histologic variants, digital pathology biomarkers, and validated molecular biomarkers associated with risk- and context-dependent outcomes 4. Encourages designing specific trials that are based on different clinical phenotypes defined by the location and distribution of radiographic metastases for which specific therapies have formal indications or exclusions
Treatment: Defining dose, schedule, toxicity, and pharmacodynamic markers
1. Encourages the use of pharmacodynamic and safety outcome measures that confirm the mechanism of action and patient benefits *v* risks and determines an optimal dose and schedule specific to the effect of a particular agent on the malignant process 2. Advises that the post-treatment biomarker measurements used to assess antitumor activity be tailored to each agent's mechanism of action and that these measurements be performed at fixed intervals (Table 4)
Baseline disease assessments
1. Expands baseline assessments to include tumor histology; the timing, duration, and response (if available) for all prior systemic treatments; a standardized assessment of blood-based, PRO-based (Appendix Table A2), and imaging-based biomarkers; prognostic risk (Appendix Table A3); and the molecular characterization of the tumor (Tables 2 and 3) 2. Emphasizes molecular/biological subtypes of prostate cancer in addition to the five clinical subtypes (defined by extent and location of metastases) 3. Defines the type of progression at trial entry as PSA-only progression, radiographic progression by site of disease spread and type of imaging, or both; for radiographic progression, records whether progression was caused by growth of existing lesions, appearance of new lesions, or both
Measuring outcomes and reporting: Blood-based and molecular measures
1. When there are progressing lesions, recommends rebiopsy of the progressing metastatic site for histology and biomarker assessment where safe/feasible or liquid biopsy 2. Suggests that PSA outcomes and progression criteria should be interpreted and redefined within the context of a drug's mechanism of action and that the anticipated timing of a potential favorable/unfavorable effect on PSA be considered and the known disconnect between imaging and PSA levels in the context of potent AR inhibition (Table 5) 3. Includes suggestions on how to define and report outcomes related to PSA declines, liquid biopsies (CTCs, ctDNA content), and serial genotyping (Appendix 2)
Measuring outcomes and reporting: PROs
1. Recognizes the importance of the patient perspective in prostate cancer clinical trials and the need to further optimize the assessment, collection, analysis, and presentation of PRO data 2. Recommends measuring disease-related symptoms including pain intensity and interference, and physical functioning, using validated instruments (Appendix Table A2) 3. Recommends collecting patient-reported adverse events using the NCI's PRO-CTCAE.
Measuring outcomes and reporting: Imaging and clinical measures
1. Expands response and progression criteria to earlier disease states prior to progression on hormonal therapy and in the nonmetastatic or PET-only setting (Table 6) 2. Expands response and progression criteria to include PSMA-PET–based imaging characteristics as a proposal to test and validate in prospective phase III trial contexts (Table 6) 3. Affirms PCWG3-based CT soft tissue and bone scan response and progression criteria as validated measures associated with survival in prehormonal and hormonal therapy–resistant contexts 4. Highlights and defines the bone-related outcomes, skeletal-related events, and symptomatic skeletal events, but suggests focusing on the latter, which represents a more direct clinical benefit to patients 5. Affirms the concept of treatment beyond progression where clinical benefit by one or more disease manifestations is being observed, thus defining an objective of NLCB

Abbreviations: ADT, androgen deprivation therapy; APMN/S/R, androgen pathway modulation–naïve/sensitive/resistant; ARPI, androgen receptor pathway inhibitor; CRPC, castration-resistant prostate cancer; CSPC, castration-sensitive prostate cancer; CT, computed tomography; CTC, circulating tumor cell; ctDNA, circulating tumor DNA; FDA BEST, US Food and Drug Administration Biomarkers, Endpoints and other Tools; NCI, National Cancer Institute; NLCB, no longer clinically benefiting; PCWG4, Prostate Cancer Clinical Trials Working Group 4; PET, positron emission tomography; PRO, patient-reported outcome; PRO-CTCAE, Patient-Reported Outcomes version of the Common Terminology Criteria for Adverse Events; PSA, prostate-specific antigen; PSMA, prostate-specific membrane antigen.

To align with a changing environment, PCWG4 presents a revised clinical model (Fig 1, Appendix Table A1, online only) to simplify the consideration of the clinical scenario or treatment indication based on previous therapies received, disease extent (localized/regional *v* metastatic), imaging modality, and genotype/phenotype considerations. We recommend avoiding the terms hormone- or castrate-sensitive *prior to* treatment given that not all patients respond similarly to hormonal therapies. Androgen pathway modulation (APM)–naïve/APM-sensitive (APMN and APMS) disease based on previous exposure and response and APM-resistant (APMR) disease are preferred terms in references to androgen deprivation therapy (ADT) and/or androgen receptor pathway inhibitors (ARPIs), favored over castration-sensitive or castration-resistant disease from a patient-centric perspective. The revised nomenclature replaces *state* with an *indication* that represents an unmet need for the patient population that would be enrolled in a specific trial. Positron emission tomography (PET)–specific staging terminology is recommended (Prostate Cancer Molecular Imaging Standardized Evaluation Framework Including Response Evaluation [PROMISE] v2.0).^12^

## ELIGIBILITY: PATIENT-CENTRIC APPROACH

### Enrollment Objectives

#### Overview

The principal objective of a clinical research study is to evaluate a specific population defined to be eligible by shared characteristics for a specific outcome/end point. For interventional therapeutic trials, the eligible population is defined by the clinical indication. We devote separate sections below to elaborate further on imaging, molecular characterization, and previous treatment. This section and Appendix 2 (online only) cover general aspects of eligibility, focusing on the patient-centric considerations.

### Baseline Measures

#### Defining the Indication

Protocol eligibility fundamentally depends on accurately determining the clinical disease characteristics of the patient population for the planned clinical indication. These characteristics continue to evolve in the classification of de novo versus metachronous (relapsed) disease, metastatic versus nonmetastatic disease defined by imaging modality, and exposure and sensitivity to hormonal therapies. Assessment must consider initial disease characteristics, patient symptoms, patterns of metastasis, previous therapies, histologic and molecular characteristics, and imaging type for optimal patient classification.

Patient experience and functional status are central to therapeutic goals in prostate cancer trials. PCWG4 recognizes patient-reported outcomes as key measures of clinical benefit, particularly for symptomatic improvement. Symptom control and preservation of functionality are important therapeutic goals that align with patient priorities. Many comprehensive and validated evaluation tools to assess symptom burden, physical findings, treatment toxicity, prognosis, and overall patient experience are available depending on the context of use (COU; Appendix Table A2). To minimize participant burden and provide the most meaningful data and relevance, assessments should focus on the investigational agent's profile and on domains most likely affected by specific treatment toxicity as compared with disease-related symptoms. We provide a summary of recommended eligibility criteria and baseline data capture for prospective clinical trials in Appendix 2 and Table 2, which will yield clinically meaningful outcomes.

**TABLE 2.** Standard Baseline Disease Assessments Recommended by PCWG4 Compared With PCWG2 and PCWG3 Recommendations

Assessment	PCWG2 (2008)	PCWG3 (2015)	PCWG4 (2026)
Histology	Not addressed	Adenocarcinoma	Retain recommendation
		Adenocarcinoma with small cell or neuroendocrine features	Retain recommendation—Clarify consensus criteria for defining variable (36) (morphology, immunohistochemistry, molecular)
		Small cell carcinoma	Retain recommendation—Clarify whether de novo pure small cell or treatment-emergent small cell
		Report Gleason sum for primary	Retain recommendation, report Grade Group
		Not addressed	Intraductal/ductal and other rare histologies
		Consider rebiopsy of metastatic disease	Retain recommendation—Modify from consider to recommended at times of treatment transition/progression. For rebiopsy in the metastatic ADT-resistant setting, consider utilizing the recently published framework for characterizing morphology and IHC attributes^13^
Clinical	History and physical examination	Age, pain, analgesic consumption, performance status, comorbidity assessment, history, and physical examination; prior local therapy; TNM stage at diagnosis; and PSA	Recommend standardized metrics for pain assessment (Brief Pain Inventory; PRO CTCAE) where relevant Recommend standardized metrics for analgesic consumption (WHO Analgesic Ladder) where relevant Recommend broader eligibility for performance status to align with FDA metrics ECOG 0-3 Specifications around prior local therapy, surgery, radiation, and intention of treatment Clarity around TNM staging regarding how this was defined clinically and pathologically and based on what imaging modality (CT/MRI/BS *v* PSMA-PET)
Prior systemic treatment	Pre- and postchemotherapy	Record each line of systemic therapy (single agent or combination) in order of administration, including start and stop dates, dose(s) and schedule(s), the disease state in which it was administered, and response (resistant *v* sensitive) on the basis of PSA if appropriate	Clarity around disease context in which treatment was administered (neoadjuvant, adjuvant, BCR, prior ADT/ARPI, metastatic disease setting, with intention of treatment, whether or not as part of definitive treatment) Clarity around treatment exposure and reason for treatment discontinuation (treatment complete, clinical/PSA/radiographic progression, toxicity)
		Record type of progression on prior therapy (PSA, radiographic [bone, nodal, visceral], clinical [eg, pain escalation])	Retain recommendation with distinction of visceral by organ (liver, lungs, CNS, other soft tissue excluding LN)
Prior radiation therapy	Not addressed	Site, administered dose per fraction, and treatment duration	Retain recommendation, include metastasis-directed RT sites
Blood-based (consider renaming laboratory parameters) biomarkers	PSA testosterone	Host: CBC with differential, ALK, kidney/liver function, albumin, LDH, testosterone^a^	Retain recommendation
		Tumor: PSA and PSA kinetics	Retain recommendation depending on context and disease state. Minimum baseline PSA to assess for PSA response end point is 1.0 ng/mL, with higher levels to be considered depending on mechanism and approach
		Optional: CEA, chromogranin A, neuron-specific enolase, CTC enumeration	Retain recommendation; CTC and ctDNA quantitation and characterization
Molecular biomarkers	Not addressed	Not addressed	Define disease state by the presence of TSG alteration, AR alterations, DNA repair alteration, SPOP, MSI-high/MSS status, and other NGS pathogenic findings Report validated genomic or digital pathology AI biomarkers when relevant Capture serial molecular profiling data, consider ancestral genotype Capture timepoint of specimen collection, type of collection (blood including circulating tumor cells or ctDNA *v* tissue), therapies administered prior to collection, and assay used with assay version date in contexts where assays may add value beyond PSA Retain raw NGS data from germline and tumor testing for future research where possible
Imaging			
Prostate/prostate bed	MRI	Retained, cross-sectional imaging of prostate region if applicable	Retain recommendation
	PSMA-PET	Not addressed	Documentation of the presence of PSMA-positive disease (all sites)
Nodal	CT: Only nodes ≥2 cm were assessed for change in size	CT or MRI: Nodes ≥1.5 cm in the short axis are considered measurable; nodes ≥1.0 cm and <1.5 cm in the short axis are considered pathologic according to clinical discretion, and nontarget; nodes <1.0 cm in the short axis are considered nonpathologic	Retain recommendation (documentation of the number of metastatic sites and categorization)
		Record pelvic and extrapelvic (retroperitoneal, mediastinal, thoracic, other) nodal disease separately; up to five nodes in total	Retain recommendation (up to five are based on RECIST)
		Record new lesions *v* growth of pre-existing lesions, and sites of new lesions	Retain recommendation; document form of progression (PSA, radiographic, symptoms) and form of radiographic progression (nodal disease, bone, visceral lung, liver, other)
	PSMA-PET	Not addressed	Documentation of the presence of PSMA-positive disease
Visceral	CT: Reported as visceral per RECIST	CT or MRI	Retain recommendation (report the number of metastatic sites)
		Record individual sites of spread (lungs, liver, adrenal, CNS) separately; up to five lesions per site	Retain recommendation (up to five are based on RECIST)
		Lesions ≥1.0 cm in the longest dimension are considered measurable	Retain recommendation
		Record new lesions *v* growth of pre-existing lesions, and sites of new lesions	Clarify the location of such lesions
	PSMA-PET	Not addressed	Documentation of the presence of PSMA-positive and PSMA-discordant disease; PSMA-avid lesions are defined by uptake, size, location, and a pattern consistent with metastatic prostate cancer to ensure high specificity
Bone	^99m^Tc MDP	Record new lesions and sites of new lesions	Retain recommendation (documentation of the number of metastatic sites)
	PSMA-PET	Not addressed	Documentation of the presence of PSMA-positive and PSMA-discordant disease
Patient-reported outcomes	None	Pain assessment, opiate analgesia consumption, physical functioning (functional status), health-related quality of life; consider fatigue, and PRO-CTCAE. Validated patient-reported outcome instruments strongly recommended	Retain recommendation and recommend use of validated instruments to assess such measures. Also, recommend focused inclusion of domains most likely impacted by specific treatment toxicity

Abbreviations: ADT/ARPI, androgen deprivation therapy/androgen receptor pathway inhibitor; AI, artificial intelligence; ALK, alkaline phosphatase; BCR, biochemical recurrence; BS, bone scan; CEA, carcinoembryonic antigen; CT, computed tomography; CTCs, circulating tumor cells; ctDNA, circulating tumor DNA; ECOG, Eastern Cooperative Oncology Group; FDA, US Food and Health Administration; IHC, immunohistochemistry; LDH, lactate dehydrogenase; LN, lymph node; MRI, magnetic resonance imaging; MSI, microsatellite instability; MSS, microsatellite stable; NGS, next-generation sequencing; PCWG2/3/4, Prostate Cancer Clinical Trials Working Group 2/3/4; PET, positron emission tomography; PRO-CTCAE, Patient-Reported Outcomes version of the Common Terminology Criteria for Adverse Events; PSA, prostate-specific antigen; PSMA, prostate-specific membrane antigen; SPOP, speckle-type POZ protein; ^99m^Tc MDP, ^99m^Tc methylene diphosphonate; TSG, tumor suppressor gene.

^a^ Ultrasensitive testosterone measures may be indicated where appropriate on the basis of the drug under study and context.

#### Blood-Based and Tissue Biomarkers

Standard laboratory assessments, including hematologic, hepatic, and renal function tests, are essential for safety and prognostic evaluation (Table 2). For testosterone assessment, PCWG4 maintains the testosterone suppression definition as ≤50 ng/dL (nmol/L). Baseline requirements should be contextualized for trials where APM might have been initiated before enrollment. Prostate-specific antigen (PSA) evaluation at baseline is required across trials, with context-specific considerations for specific minimum thresholds such as treatment-naïve biochemical recurrence-only trials.

Prostate cancer is a biologically heterogeneous disease with diverse drivers that affect patient prognosis and response to therapies, so baseline molecular characterization should help guide trial selection and biomarker development.^14^ Much of this heterogeneity not only exists within patients and tumors but also changes over time with treatment. Thus, in addition to the collection of established baseline prognostic factors in all phase II and phase III clinical trials (Appendix Table A3),^15-18^ we also advocate for serial collection of biospecimens, conforming to the ASCO biopsy framework.^19^ Serial collection of whole blood and plasma is minimally invasive and is strongly encouraged, and tissue collections should be considered when safe, feasible, and scientifically justified.

#### Progression Criteria for Enrollment in Clinical Trials

For clinical trial evaluation, criteria for progression must be clearly defined and the form of progression per patient must be collected (imaging, PSA, symptoms). PCWG4 maintains its criteria based on PSA (biochemical) or radiographic progression (Table 3). For patients on ADT/ARPI therapy, any confirmed PSA rise may indicate progression; however, radiographic progression can occur without PSA elevation or symptoms. Radiographic progression should be characterized by site of tumor involvement (lymph node, bone, visceral lungs/liver/other, prostate/prostate bed) and imaging modality, noting whether progression involves existing measurable lesions, new lesions, or both. Contemporary protocols might include patients with disease detectable only by prostate-specific membrane antigen (PSMA)-PET. Progression should be defined by new lesions rather than by criteria based on standardized uptake value (SUV) and should ideally be compared with a previous similar PSMA-PET as an appropriate contemporary comparator. PCWG4 urges a flexible definition in which new lesions should be called only if there is high reader confidence based on a combination of factors including intensity of PSMA uptake above blood pool, anatomic correlates on cross-sectional imaging, and pattern of spread. This practical definition of new lesions for progression is consistent with how PCWG3 approached defining bone metastases on scintigraphy. Thus, to avoid premature designation of progression, radiographic interpreters should have a high degree of clinical confidence that a new site of PSMA uptake indeed represents progressive disease.

**TABLE 3.** Recording of Disease and Progression at Trial Entry by Disease Manifestation

Variable	PCWG2 (2008)	PCWG3 (2015)	PCWG4 (2026)
Blood-based			
PSA	Obtain the sequence of rising values at a minimum of 1-week intervals	Retained	Retained
	2.0 ng/mL minimal starting value	1.0 ng/mL is the minimal starting value if confirmed rise is only indication of progression unless pure small cell carcinoma	Record PSA at baseline. No minimum required but depends on disease state and trial objectives. Confirmed rise needed if only indication of progression unless pure small cell carcinoma. Minimum baseline PSA to assess for PSA response end point is 1.0 ng/mL, with higher levels to be considered depending on the mechanism and approach
	Estimate pretherapy PSADT if at least three values are available ≥4 weeks apart	Retained	
Imaging (CT or MRI)			
Nodes	Nodal progression sufficient for trial entry independent of PSA	Retained	Retained For PSMA-PET: Two new PSMA-avid lesions, for appropriate studies
	Measurable lesions not required for entry	Retained	Retained
	Use RECIST to record nodal lesions as target or nontarget	Modified RECIST 1.1 criteria, separate pelvic and extrapelvic disease, up to five nodal lesions total recorded	Retained For PSMA-PET: Track both PSMA-avid and nonavid lesions
	Only lymph nodes ≥2 cm in diameter (long axis) were actionable as progressive disease	Previously normal (1.0-cm) lymph nodes must have grown by ≥5 mm in the short axis from baseline or nadir and be ≥1.0 cm in the short axis to be considered to have progressed If the node progresses to ≥1.5 cm in the short axis, it is measurable; nodes that have progressed to 1.0 to <1.5 cm are pathologic, subject to clinical discretion and nonmeasurable For existing pathologic adenopathy, progression is defined per RECIST 1.1	Retained
	Record the presence of nodal and/or visceral disease separately	Retained with modification Nodal sites: Locoregional: Pelvic only Extrapelvic: Retroperitoneal, mediastinal, thoracic, or other	Retained For PSMA-PET: Both PSMA-avid and nonavid lesions should be separately tracked
Viscera	Visceral progression sufficient for trial entry independent of PSA	Retained but recorded separately by site of spread (lungs, liver, adrenal, CNS); up to five lesions per site of spread	Retained For PSMA-PET: Both PSMA-avid and nonavid lesions should be separately tracked
	Measurable lesions not required for entry	Retained	Retained For PSMA-PET: As above
	Use RECIST to record visceral lesions as target or nontarget	Retained	Retained For PSMA-PET: As above
	Record the presence of nodal and/or visceral disease separately	Retained with modification Visceral sites: Lungs, liver, adrenal, CNS	Retained For PSMA-PET: As above
Prostate/prostate bed (primary site)	Record previous treatment of primary tumor	Retained	Retained
	Perform-directed pelvic imaging (CT, MRI, PET/CT, transrectal ultrasound) to document the presence or absence of disease	Retained	Retained For PSMA-PET: As above
Bone	Two new lesions	Retained	Retained For PSMA-PET (for appropriate studies): Two new PSMA-avid lesions
	Confirm ambiguous results by other imaging modalities (eg, CT or MRI)	Retained, but only positivity on the bone scan defines metastatic disease to bone	Retained For studies that allow for PET-defined lesions only (independent of conventional imaging modality findings), PET findings must independently have a high level of confidence of positivity
Other sites of disease	Patients with treated epidural lesions and no other epidural progression are eligible	Retained	Retained
Type of progression at trial entry			
	Not addressed	Report separately: PSA only Bone only and with or without nodal disease Nodal disease only (no bone disease present) Visceral (lungs, liver, adrenal, CNS) disease (6 other sites) Record new lesions and site of new lesions v growth of preexisting lesions, or both	Retained For PSMA-PET: Track avid and nonavid lesions independently
Other markers			
Patient-reported outcomes	Not addressed	For pain palliation analyses, the presence of clinically meaningful pain at baseline (eg, ≥4 on a 10-point pain intensity scale) is a prerequisite; for pain progression analyses, patients may have any level of pain at baseline, including no pain	Retained

Abbreviations: CT, computed tomography; MRI, magnetic resonance imaging; PCWG2/3/4, Prostate Cancer Clinical Trials Working Group 2/3/4; PET, positron emission tomography; PSA, prostate-specific antigen; PSADT, PSA doubling time; PSMA, prostate-specific membrane antigen.

## BASELINE IMAGING

### Overall Approach to Imaging

PCWG3-defined radiographic progression-free survival (rPFS) has proven to be a robust imaging end point across multiple phase III prostate cancer trials and across a range of disease states, where the moderate to strong association between rPFS and overall survival (OS) has been maintained across clinical trials.^20-25^ This definition has been feasible to incorporate in large international studies. rPFS has historically been treated by regulatory agencies as an intermediate end point, whereas metastasis-free survival (MFS) by computed tomography (CT)/bone scan/magnetic resonance imaging (MRI) has been treated as a de facto clinical event.^26,27^

PCWG4 considers PSMA-PET as noninvestigational for staging and for demonstrating distribution of disease in relapsed patients as PSMA-PET has regulatory recognition for these indications. However, PSMA-PET is investigational for determination of treatment response and progression.^1,2,30,31^ Fluorodeoxyglucose (FDG)-PET may be used in specific circumstances to identify non–PSMA-avid lesions for both adenocarcinoma and cancers on the neuroendocrine spectrum.^33^

A number of studies have promoted standardization of PSMA-PET clinical reporting.^12,34-36^ Standard assessment criteria for positive lesions include any site with uptake above physiologic background levels.^37^ More recently, given reasonable reproducibility of PET quantitative metrics in reference organs such as the blood pool and liver, using blood pool as the common reference region for both ^18^F DCFPyL and ^68^Ga–PSMA-11 has been proposed.^13,34^ In the setting of ADT-resistant and metastatic prostate cancer, clinical PSMA-PET reporting has been largely focused on determining patient eligibility for ^177^Lu PSMA therapy rather than TNM stage.^3,38-40^ Additional recommendations to capture heterogeneous PSMA expression in a straightforward manner in clinical reports include providing the maximum SUV of the tumor sites with highest and lowest uptake.^12^ Standardizing clinical reporting of response assessment is increasingly important. Several proposals and an expert consensus statement have been published given the anticipated increasing use of PSMA-PET for response assessment.^12,35,41,42^ The focus of PCWG4 is not reporting, as consensus frameworks such as SPARC, PROMISE, and others^36,43,44^ provide nomenclature and sufficient clinical guidance for implementation, but rather to establish standardized trial methodologies for incorporating PSMA-PET into prostate cancer research studies, especially as outcome measures.

### Baseline Imaging

PCWG4 recommends that pretreatment imaging in research studies include PSMA-PET/CT imaging where feasible and covered by either insurance or the research. PSMA imaging is particularly important. A clinical trial may consider patients by the distribution exclusively by PSMA-PET,^44^ exclusively by previous standard imaging using CT/MRI and bone scan, or by both. However, all three sets of imaging modalities (PSMA-PET, bone scan, and CT or MRI) should be obtained before study entry and independently recorded when feasible. PCWG3 recommended that CT/MRI should be obtained with contrast; this position is maintained by PCWG4, even if the patient undergoes PSMA-PET, as the tracer does not substitute for IV contrast. Many centers can perform a contrast-enhanced CT in conjunction with the PET, which adds to patient convenience.

## INTERVENTION

In PCWG4, the term intervention refers not only to therapeutic interventions but also to biomarkers or behavioral/lifestyle interventions critical for determining dose, schedule, and antitumor mechanism or efficacy/safety of therapeutic interventions.

PCWG4 affirms that early-phase (phase I/II) interventional clinical trials need to include appropriate-dose optimization strategies that culminate in the most appropriate dose for phase III testing. PCWG4 endorses FDA Project Optimus^45^ within the Oncology Center of Excellence to reform the dose optimization and dose selection paradigm.^46^ The maximum tolerated dose should not necessarily equate to the recommended phase II (or phase III) dose. In addition to tolerability and safety, the dosing schedule should be based on the mechanism of action, target engagement, pharmacokinetics, and drug interactions in representative patient populations. Biomarker (nontherapeutic) interventions that are undergoing clinical validation in a particular COU should be evaluated with comparable scientific rigor. Thus, a goal of PCWG4 is the development and incorporation of tools for on-treatment biomarkers, liquid and imaging biomarkers, in particular, that have completed analytical validation, with qualification of laboratory biomarkers within Clinical Laboratory Improvement Amendments standards.^47^

PCWG4 recognizes that the timing and cadence of on-treatment assessments, which are themselves interventions as noted, should be tailored to the disease state, therapeutic mechanism of action, and hypothesized rate of change in the biomarker/assessment. Suggested cadence for liquid and imaging biomarkers is given in Tables 4 and 5 in the APMN/S and APMR settings.

**TABLE 4.** PCWG4 Suggested Testing Intervals Based on the Disease State

Imaging	Baseline	First 6 months	After 6 months	Rising PSA or Early Evidence of Radiographic Disease or Clinical Progression
Neoadjuvant/adjuvant (MFS end point)				
Bone scan and CT	Yes	At 6 months	Every 6 months	Imaging frequency may be increased to document disease recurrence
PSMA	Yes	At 6 months	At 12 and 18 months	Imaging frequency may be increased to document disease recurrence
APMS/N, biochemical recurrence with/without metastases (MFS or rPFS end point)				
Bone scan and CT	Yes	At 3 and 6 months	Every 6 months	Imaging frequency may be increased to document disease progression
PSMA	Yes	At 3 and 6 months	At 12 and 18 months	Imaging frequency may be increased to document disease recurrence
APMR (ADT and/or ARPI) with/without metastases (MFS or rPFS end point)				
Bone scan and CT	Yes	Per PCWG3	Every 3 months	Imaging frequency may be increased to document disease progression
PSMA	Yes	For the first 3 scans	Every 6 months	Imaging frequency may be increased to document disease progression

NOTE. To minimize patient burden, assessments after/during therapeutic intervention should occur in concordance with other clinical visits when possible.

Abbreviations: ADT, androgen deprivation therapy; APMS/N, androgen pathway modulator–sensitive/naïve; APMR, androgen pathway modulator resistant; ARPI, androgen receptor pathway inhibitor; CT, computed tomography; MFS, metastasis-free survival; PCWG3/4, Prostate Cancer Clinical Trials Working Group 3/4; PSA, prostate-specific antigen; PSMA, prostate-specific membrane antigen; rPFS, radiographic progression-free survival.

**TABLE 5.** Definitions of Secondary Delay/Prevent End Points (other than imaging-based end points)

Measure	Baseline Assessment	Frequency of Assessment	Definition of Progression
Clinical and symptom-based markers			
NLCB	No baseline assessment	Continuous	Time to clinical deterioration (eg, weight loss due to disease, pain progression due to disease, functional deterioration not due to toxicity) that cannot be addressed by local treatment to a single or a small number of progressive lesions
Symptoms, pain, and QOL	Record at baseline, preferably using PROs	Every 3-6 weeks for symptoms/pain; every 8-12 weeks for QOL	Time to deterioration of disease-related symptoms, cancer-related pain, or patient-assessed QOL
PFS2	No baseline assessment	Imaging assessments for PFS1 are described by Group 4; imaging assessments for PFS2 should be every 8-12 weeks	Time interval from initial random assignment to the time of second radiographic or clinical progression following crossover; time from crossover therapy (PFS1) to second progression should also be recorded
Time to next therapy	No baseline assessment	Not applicable	Time to initiation of next systemic therapy
Overall survival	No baseline assessment	Continuous	Time to death from any cause; record cause of death
Prostate cancer–specific survival	No baseline assessment	Continuous	Time to death from prostate cancer (with censoring of non–prostate cancer-related deaths)
Blood-based markers			
PSA	Record the PSA level at baseline. No minimum value required	Every 3-6 weeks depending on context	PCWG2/3 criteria. In the setting of APM, also record time to *any* increase in PSA level from baseline/nadir, confirmed by one additional rising PSA measurement and with a minimum absolute increase of 0.2 ng/mL.^3^ Transient rises followed by subsequent PSA declines while maintaining therapy (flare) should be recorded and would not define PSA progression
Alkaline phosphatase, LDH	Record alkaline phosphatase, LDH levels at baseline	Every 3-6 weeks	Time to *any* increase in ALK/LDH from baseline/nadir, confirmed by one additional rising measurement
Serum chemistry, CBC	Record serum chemistries, CBC parameters at baseline	Every 3-6 weeks	Time to *any* increase in a chemistry/CBC parameter, confirmed by one additional rising measurement
Molecular markers			
CTC enumeration	Record the number of CTCs at baseline	Every 8-12 weeks	Time to *any* increase in CTC count from baseline/nadir
ctDNA concentration and/or tumor fraction	Record the presence or absence, and concentration of ctDNA at baseline	Every 8-12 weeks	Time to *any* increase in tumor fraction from baseline/nadir
RNA/DNA alterations	Record the presence or absence of the alteration at baseline	Every 8-12 weeks	Time to first detection of a particular molecular alteration, if not present at baseline

Abbreviations: ALK, anaplastic lymphoma kinase; APM, androgen pathway modulation; CTC, circulating tumor cell; ctDNA, circulating tumor DNA; LDH, lactate dehydrogenase; NLCB, no longer clinically benefiting; PFS1, first progression-free survival; PFS2, second progression-free survival; PROs, patient-reported outcomes; PSA, prostate-specific antigen; QOL, quality of life.

## OUTCOMES: RESPONSE INDICATORS

### Response Assessment

Development of response biomarkers is a high priority for advancing prostate cancer care. The PCWG4 committee encourages the incorporation of novel biomarkers for this purpose following the FDA's BEST criteria.^48^ A qualified biomarker must be subject to a specific interpretation and application within a COU. PCWG4 emphasizes that the COU for prostate cancer should apply to a given disease state or indication that strongly correlates with survival or other measures of clinical benefit and that prospective inclusion of biomarkers in trial design is preferred to inform treatment selection or change or to provide a more rapid assessment of efficacy.

At this time, only four blood- or tissue-based response biomarkers are considered by this group to be near-term candidates to be formally developed as qualified biomarkers: pathologic response in neoadjuvant therapy, PSA declines/nadir in specific contexts, zero detectable circulating tumor cells (CTC0) in metastatic disease, and circulating tumor DNA (ctDNA) response in metastatic disease. However, these response biomarkers are not yet established as surrogates of OS or patient benefit for regulatory approval. See Appendix 2.

### Response Assessments: CT/MRI and Bone Scintigraphy

For cross-sectional soft tissue and bone imaging, PCWG3 definitions of response apply for those patients with measurable disease. As was true with PCWG3, on-treatment alterations demonstrated by cross-sectional imaging should not be merged with those seen on bone scintigraphy. Changes in cross-sectional imaging should be reported separately from bone scintigraphy and not combined into grouped response/progression criteria.

### Response Assessment: PSMA-PET

PSMA-PET enables quantification of lesional radiotracer uptake and correlation with imaging CT/MRI and therefore could reasonably allow for descriptions of on-treatment changes other than progression, such as response. Schemas for assessing progression and response by PSMA-PET have been introduced by other consensus groups.^35,41^ They may differ from PCWG4 criteria, which follow a specific set of principles, with criteria that are broadly and internationally applicable at all sites performing clinical trials, without the need for specialized or proprietary software or hardware. Furthermore, while many PET parameters can be measured, these measures are not yet clinically qualified as biomarkers and, therefore, should be considered investigational. For these reasons, PCWG4 does not incorporate changes in PET SUV or volume as proof of progressive disease or response.

Changes in SUVmax, mean, or SUV-assessed tumor volume remain investigational, requiring further validation and clinical credentialing. Nevertheless, we highly recommend the collection of serial PSMA-PET images ideally using the same tracer and imaging protocol as part of all clinical trials for future analysis and validation of potential response criteria. We further recommend documenting complete responses (CRs) by PSMA-PET as the complete resolution of PSMA-positive disease is a binary event, does not require specialized software, and is recordable. Resolution is defined as uptake below blood pool by visual assessment. Sclerotic bony lesions do not need to resolve on CT for a CR to be documented. A PSMA-PET complete response should be recorded separately from RECIST anatomic reads (ie, PSMA-PET findings may resolve, but the patient could independently have progressive disease by RECIST) as a PSMA-PET CR may be either demonstrative of prostate cancer cell death or of dedifferentiation, perhaps to a more biologically aggressive state. There will be no PSMA-PET partial response or stable disease category. Progression in bone by PSMA and by bone scintigraphy will follow the same criteria as defined for on-study enrollment, described above. Lesions that were present at the treatment start, fully resolve on PET during treatment to below blood pool, and then subsequently reappear are considered new lesions for determining progression and should be recorded separately as recurrent disease. Table 6 summarizes PCWG4-defined progression criteria by PSMA-PET imaging in the APMS and APMR settings for future validation in prospective trials.

**TABLE 6.** Synthesis of Delay/Prevent Outcomes by Imaging Modality

Imaging Modality	Pretreatment Scan	On-Treatment Scan 1 (≥week 8)	On-Treatment Scan 2	All Subsequent Scans
Bone scintigraphy				
	Comparator for on-treatment scan 1	Comparator for all subsequent scans POD only if ≥2 new lesions confirmed on the subsequent scan (2 + 2)^a^	POD: ≤5 new lesions: PCWG3 criteria 2 new lesions that are confirmed on a subsequent scan (2 + 0)^a^ Or ≥6 new lesions	Same as on-treatment scan 2
CT/MRI				
Any measurable disease	Comparator	PCWG3/RECIST	PCWG3/RECIST	PCWG3/RECIST
PSMA-PET				
Bones and lymph nodes and lung metastases (non-RECIST qualifying by + on PET only)	Comparator	POD: ≤5 new lesions: 2 new lesions that are confirmed on a subsequent scan ≥6 new lesions	Same as on-treatment scan 1	Same as on-treatment scan 1
Liver parenchyma and nonpulmonary viscera	Comparator	POD: Any single new lesion that represents disease	Same as on-treatment scan 1	Same as on-treatment scan 1

NOTE. When new lesions are accounted for by PSMA-PET (eg, ≥6), bone, nodes, and lung metastases are aggregated. The pretreatment PET/CT is the comparator. Imaging-specific minimum criteria proposed for rPFS end points for CT, MRI, bone scan, and PSMA-PET imaging. PSMA-PET scans performed earlier than 8 weeks after treatment start will be ignored for interpreting progression of disease. New lesions include both newly seen and newly recurrent for those lesions that were in a previous complete remission.

Abbreviations: CT, computed tomography; MRI, magnetic resonance imaging; PCWG3, Prostate Cancer Clinical Trials Working Group 3; PET, positron emission tomography; POD, progression of disease; PSMA, prostate-specific membrane antigen; rPFS, radiographic progression-free survival.

^a^ The date of POD is the earliest time point of imaging-based progression or death, not the confirmatory scan date. Patients who develop any liver, adrenal, pleural, and other nonpulmonary metastases will be declared as progressing even if they have a single metastatic focus relative to the pretreatment scan.

### Response Assessment: Patient-Reported Outcomes

We encourage investigators to engage with regulatory authorities early in trial design to discuss the best and most parsimonious strategy for collection of patient-reported outcomes measures (PROMs) for a given study based on the anticipated impacts on disease burden symptoms and toxicities while minimizing patient survey burden.^49-51^ The preferred PROM inventory, domain of interest, and monitoring schedule may differ by disease state (eg, localized or advanced prostate cancer), treatment modality, or expected side effects. In some situations, it may be appropriate to select a PROM of a key symptom as the coprimary or secondary end point for a clinical trial (Appendix Table A2), along with objective tumor response assessments. A pre-existing hypothesis or conceptual framework must guide the selection of the appropriate PROM.

Resources are now available to help investigators improve the scientific rigor for PROM incorporation into protocols and also to aid in matching a particular quality-of-life (QOL) objective (including domain and timeframe of interest) with appropriate statistical methodology.^52,53^ Investigators are recommended to consider presenting the probability of achieving a minimal clinically important difference and to use time-to-event analyses when reporting QOL and symptom data.^54^ We highlight the newer resources that have become available since the publication of PCWG3 in 2016. Inclusion of such data at the time of initial trial reporting more fully frames the potential risks and benefits for any given therapy. Moving forward, national and international regulatory agencies have indicated that the patient experience will factor prominently in decision making.^55^

### Response Assessment: Biomarkers

Four strongly prognostic intermediate response end points including post-treatment PSA changes, pathologic response, circulating tumor cell changes, and ctDNA changes are discussed in detail in Appendix 2. These end points may serve as phase I to II trial signals of efficacy to inform next steps in clinical development depending on COU.

## OUTCOMES: DELAY/PREVENT

When deciding whether a patient is still benefiting from an ongoing therapy, three areas should be considered: (1) imaging-based definitions of disease progression, (2) clinical definitions of progression, and (3) changes in circulating biomarkers (eg, PSA, others), summarized in Tables 5 and 6. PCWG4 recommends reporting the type of progression (PSA, imaging, death, symptoms, other biomarkers) and dates. Timelines for progression should not be reset for treatment interruptions or the use of metastasis-directed therapy (MDT), but these treatment breaks, eugonadal periods, and use of subsequent MDT should be recorded. PCWG4 recommends reporting of symptomatic skeletal event-free survival (SSEFS) similarly to PCWG3 and distinct from imaging-based response/delay end points given that these events often do not meet RECIST or imaging-based progression criteria. Finally, imaging-based pseudoprogression^30,56,57^ is defined as new lesions by any modality that are unconfirmed, typically in the context of an otherwise responding patient without other manifestations of progression, and PCWG4 recommends capturing these data in clinical trials.

### Changes in Imaging

#### Bone Scintigraphy and CT/MRI

For PCWG4, there will be limited changes in the rigorously tested criteria for the interpretation of findings on bone scintigraphy and conventional cross-sectional imaging as defined in PCWG3. For patients with metastatic disease, the 2 + 2 rule will continue to apply (at least two new lesions on each of the first two on-treatment scans to qualify as progression. The first on-treatment scan will represent the date of progression when 2 + 2 criteria are met and serve as the new baseline for future scans if the 2 + 2 criteria are not met).

One significant change that will be made with PCWG4 is regarding bone imaging. Previously, if patients did not meet 2 + 2 criteria in the first two on-treatment scans, at least two new lesions confirmed on a subsequent bone scan performed at least six weeks from the prior scan (2+0) were required to meet progression criteria. In an effort to prevent deleterious disease progression while waiting for a confirmatory scan in rapidly progressive patients while meeting PCWG3 criteria,^58^ we now propose that a confirmatory scan not be required if a restaging scan describes ≥6 additional bone lesions relative to the first on-treatment scan (Table 6).^58^ Doing so avoids unnecessary waiting for a confirmatory scan if significant disease progression is observed. The date of progression is the date of documenting ≥6 lesions relative to the first on-treatment scan. This change in PCWG4 should not significantly alter the declared time of rPFS relative to PCWG3. In PCWG3, the date of progression was backdated from the confirmatory scan to the date of the scan in which two or more new lesions appeared. This would be the same date of progression in PCWG4 for a patient that has six or more new lesions, without the need for a confirmatory scan. No changes will be made for lower-volume bone disease progression (≤5 lesions) relative to PCWG3, that is, a confirmatory scan will still be required to document progression. This guideline will apply across the board for patients with metastatic APMS and APMR disease.

For those without metastatic disease, MFS is the primary validated radiographic end point using CT/MRI or bone scan.^59^ PCWG3 deferred to regulatory authorities any specific definition of newly diagnosed metastatic disease. Several registration trials in the nonmetastatic castration-resistant prostate cancer (APMR) space used similar definitions, using a combination of bone scintigraphy and cross-sectional imaging.^60-62^ PCWG4 will not change this approach, given that three drugs have already been approved using it. MFS in bone should not be defined on the first on-treatment scan when flare might occur and should therefore be defined using the first on-treatment scan as a baseline.

#### PET Imaging

PCWG4 now incorporates PSMA-PET into proposed progression criteria. On-treatment PET imaging using the same tracer as the pretreatment PET is recommended. PSMA-PET is far more specific than bone scintigraphy or CT and will detect disease earlier and with far greater accuracy. Confirmatory scans, therefore, are usually not necessary. However, consistent with the spirit of PCWG3, patients should be kept on therapy if PSMA-PET findings are of dubious clinical impact. The guidelines for declaring a radiographic progression event, therefore, distinguish between organ sites, similar to the above guidelines, and separate liver and other nonpulmonary visceral progression from nodes, bone, and lung progression. It should be noted that some therapies do modulate PSMA expression very early in the treatment course (within the first 2 months of treatment), which can confound interpretation. Therefore, early scans should not be used to define progression.

##### Definition of progression in bone.

Two or more new lesions will constitute progression in bone. PSMA lesions may or may not have an anatomic correlate. For pre-existing bony lesions that are evident only by PSMA-PET, increases in SUV measures alone, whether by mean, max, volume, or other parameters, will not constitute progression although all images should be collected for future analysis of these parameters. New lesions will not be defined solely by an SUV cutoff of above blood pool, but by the combination of uptake, size, location, and a pattern consistent with metastatic prostate cancer to ensure high specificity. Two new lesions require confirmation on a subsequent scan performed ≥6 weeks after the first scan, whereas six or more lesions do not require confirmation given the higher risks for clinical deterioration for these polymetastatic progressors.^58,63^ The pretreatment scan is the comparator. We recognize that some protocols will permit MDT to a limited number of new lesions evident by PSMA PET. Such an event should be recorded, but the progression timeline should remain unchanged if the protocol demands rPFS by standard imaging. See criteria in Table 6.

##### Definition of progression for nonbony disease.

For measurable disease, PCWG3/modified RECIST still apply to the CT/MRI component of the PSMA-PET. We recommend that the CT component of the PSMA-PET be performed with contrast (either as a separate study or in conjunction with the PSMA-PET/CT).

For PSMA-PET, nodal and pulmonary metastases will be treated similarly to bone. Changes in lesional avidity do not constitute progression. Two or more new lesions in aggregate (bone, nodes, and pulmonary metastases) will constitute progression but require confirmation unless there are six or more new lesions in which case no confirmation is needed. PET avidity should be reported separately from CT findings. Note that these criteria apply to sites of disease that otherwise are not measurable (as RECIST apply) or scintigraphically evident (as PCWG3 applies). All three measures of progression should be reported separately (Table 6) especially if there is PET-only progression and the patient remains on treatment. The pretreatment scan is the comparator.

For patients with hepatic parenchyma and other nonpulmonary visceral sites that are PSMA-avid and do not meet RECIST, any single new lesion by PSMA-PET constitutes progression (Table 6).

For those with nonmetastatic disease, MFS by PSMA-PET is defined by the appearance of a new lesion felt by uptake, size, location, and a pattern consistent with metastatic prostate cancer to ensure high specificity. In the spirit of existing MFS definitions used for approval for existing drugs, for isolated PET-evident lesions, correlation with some anatomic finding should be present.

### Clinical Definitions of Progression

#### No Longer Clinically Benefiting

No longer clinically benefiting (NLCB)^11^ is defined as clinical deterioration (eg, cancer-related weight loss, cancer-related pain progression, functional deterioration not because of toxicity) that cannot be addressed by local treatment to a single or a small number of progressive lesions. NLCB criteria should be attributed to the underlying disease, distinct from toxicity-related deterioration. The NLCB principle allows treatment beyond radiographic progression if the patient is deemed to be deriving overall benefit, despite potentially meeting radiographic progression criteria. Moreover, as long as the NLCB end point is not met, local treatment to progressive lesions can be applied,^64^ provided that the majority of disease remains controlled. Treatment beyond progression must not be pursued in the setting of overall symptomatic deterioration or broad QOL deterioration. Even if treatment in a clinical trial is continued until the NLCB end point, objective progression end points should still be documented.

Additional patient-centric delay/prevent end points are discussed in Appendix 2.

#### Overall Survival

OS (time to death from any cause) should still be reported as the gold standard delay/prevent end point in all settings. Prostate cancer–specific survival, defined as freedom from prostate cancer–related death (where deaths from non–prostate cancer-related causes are censored), can also be of value, provided that treatment-related mortality is low and not increased by the treatment under study. PCWG4 recommends that the cause of death be recorded where available.

### Changes in Circulating Biomarkers

Changes in circulating biomarkers alone should not be used as an approval end point, nor a change in treatment. Given that circulating biomarkers often fluctuate, decreases or increases should only be called after at least two consecutive confirmatory measurements are obtained. The first rise should be used to compute the time to increase of the biomarker. See Appendix 2.

#### PSA Progression

While retaining PCWG2/3 PSA progression criteria,^9,65^ we also propose a modification to the definition of PSA progression, given that with the use of next-generation AR-directed agents (APMs), for example, radiographic or clinical progression may occur before or even in the absence of PSA progression as defined in PCWG3, or before even any confirmed PSA rise.^66-69^ Thus, PCWG4 recommends simplifying PSA criteria to report PSA changes from baseline if no decline or from nadir if a decline is present. Here, we propose to define PSA progression in the setting of androgen pathway modulation as the time to *any* increase in PSA level from baseline/nadir, confirmed by one additional rising PSA measurement at least 21 days apart (the progression date is the date of the first rise). A minimum rise of 0.2 ng/mL is required given the inherent noise and variability in the PSA assay.^70^ Transient PSA rises (flare) followed by declines are observed with a range of therapies and should be recorded, and PCWG4 does not recommend a change in treatment based solely on PSA changes. If the above definition of PSA progression occurs in the initial hormone therapy setting, this equates to time-to-ADT/ARPI resistance unless these changes are transient and/or unconfirmed. To enable correlation with rPFS and OS, we recommend reporting PSA progression by both definitions.

## DISCUSSION

In conclusion, PCWG4 builds on the strong foundation of previous working groups to refine clinical trial eligibility, end points, objectives, and serial biological and patient-centric classifications of advanced prostate cancer, along with updated nomenclature to inform design of clinical trials. These best practices are recommended in conjunction with appropriate clinical trial designs in collaboration with regulatory agencies to test therapies in biomarker-defined populations while also testing safety and efficacy in clearly defined biomarker-negative subgroups to ensure the clinical validation of an analytically valid biomarker. Ultimately, these recommendations should accelerate personalized therapeutic development, maximizing survival and QOL improvements to those patients most likely to benefit.

## APPENDIX 1. PCWG4 Additional Authors: Writing Group

Joshi J. Alumkal, MD; Ana Aparicio, MD; Pedro Barata, MD, MSc; Charles Drake, MD, PhD; Julie Graff, MD; Anis A. Hamid, MBBS, PhD; Elisabeth I. Heath, MD; Jones Nauseef, MD, PhD; Daniel Lage, MD, MBA, MSc; Nadine Mallak, MD; Akash Patnaik, MD, PHD, MMSc; Kristofer Prepelica, PhD; Dana E. Rathkopf, MD; Matthew Rettig, MD; Steven P. Rowe, MD, PhD; Susan F. Slovin, MD; Mary-Ellen Taplin, MD; and Nicholas Zorko, MD, PhD

## APPENDIX 2. Eligibility

### Representation and Inclusion

Patients enrolled in clinical trials should reflect the larger population that is at risk for the medical need that the intervention is intended to treat. The demographic spectrum of patients with prostate cancer who enroll to clinical trials remains discordant from the broader population of all patients affected by prostate cancer (Balakrishnan AS, et al: J Urol 201:259-267, 2019; Lythgoe MP, et al: Prostate Cancer Prostatic Dis 24:1208-1211, 2021). These discrepancies should be rectified. Prostate Cancer Clinical Trials Working Group 4 (PCWG4) recommends investigators actively endeavor to address obstacles to participation in research studies (Ghebre RG, et al: Cancer 120:1122–1130, 2014), tailor delivery of educational content (Meropol NJ, et al: J Clin Oncol 34:469-478, 2016), and ensure a representative trial population reflective of the target at-risk population (Mahal BA, et al: Eur Urol Oncol 5:18-29, 2022). PCWG4 endorses recommendations made by the National Cancer Institute, US Food and Drug Administration (FDA), ASCO (Kim ES, et al: J Clin Oncol 35:3737-3744, 2017; FDA: https://www.fda.gov/media/178018/download, 2024), and other groups to reduce restrictions on trial eligibility and barriers to trial access (Lowder D, et al: Cancer Lett 531:71-82, 2022). We thus recommend removal of unnecessary barriers (geographic, logistic, and protocol-imposed) to better streamline protocols and encourage early partnership with community stakeholders (patient advocates and community leaders) to optimize equity. Clinical trial facilitators, including reimbursement for travel, parking, lodging, and time-off work for patients and caregivers, should be considered and provided (Nipp RD, et al: Oncologist 21:467-474, 2016). The integration of telemedicine and trial decentralization for outreach and in study design can enhance participation and accessibility and reduce costs (Guadamuz JD, et al: JCO Oncol Pract 19:1206-1214, 2023; Culli L: https://publichealth.jhu.edu/2025/bridging-the-digital-divide-in-health-care-a-new-framework-for-equity, 2025). Finally, eligibility criteria regarding chronic conditions and performance status should be more inclusive of the patient population in the real-world setting, which will not only accelerate accrual but also better reflect the actual patient population and provide information on a broader patient population that will ultimately benefit if the trial is successful (Magnuson A, et al: Clin Cancer Res 27:2424-2429, 2021).

### Histology

While adenocarcinoma remains the predominant histology in prostate cancer, awareness of variant subtypes, such as small cell and neuroendocrine prostate cancer, both of which are more aggressive and associated with inferior survival, is critical. In addition, since 2016, intraductal carcinoma has been characterized as a distinct entity by the WHO, and its presence has been associated with more aggressive disease and worse outcomes (Nelson TJ, et al: Clin Genitourin Cancer 21:452-458, 2023). Historical exclusion of small cell or neuroendocrine histologies from trials warrants reconsideration in certain contexts, particularly given the increasing recognition of lineage plasticity that may occur under continuous androgen receptor (AR)–targeted therapy pressure. Baseline tissue characterization using validated molecular, proteomic, or digital pathology biomarkers should guide trial selection. PCWG4 recommends rebiopsy of patients progressing in the androgen pathway modulator–resistant (APMR) setting and use the existing framework for characterizing morphology and immunohistochemistry attributes, including markers for AR signaling, neuroendocrine differentiation, and proliferation to classify phenotype (Haffner MC, et al: Clin Cancer Res 31:466-478, 2025). Digital pathology and multimodal artificial intelligence biomarkers can provide additional prognostic and predictive significance across a range of disease settings (Armstrong AJ, et al: J Clin Oncol 43:3494-3504, 2025; Spratt DE, et al: Res Sq rs.3.rs-2790858, 2023) where appropriate and validated.

### Previous Treatment

Previous treatment documentation should include the timing, duration, and outcomes of all systemic therapies, such as previous exposure versus progression on these therapies. PCWG4 de-emphasizes the use of lines of therapy (eg, first-line, second-line, etc) as such terminology is not specific and what constitutes a line can be confusing. Instead, the preference is to report previous exposures to specific agents or classes of agents and outcomes with those agents. Details of radiation therapy (curative, salvage, or metastasis-directed) and surgical interventions should be recorded.

### Genotype

PCWG4 recommends performing Clinical Laboratory Improvement Amendments–based panel or whole-genome sequencing for tumor somatic and germline alterations to identify actionable genomic subtypes and predict therapeutic responses, prognosis, treatment resistance over time, and cascade testing to inform cancer risk to relatives. Tumor RNA expression classifiers linked to therapeutic benefit may also serve as appropriate selection criteria when clinically validated (Hwang J, et al: Clin Cancer Res 31:936-948, 2025). Sources of tumor DNA include primary prostate tissue, metastatic biopsy, or circulating tumor DNA (ctDNA), each with advantages and limitations. Ideally, such assays should be integral to the trial design and companion development of therapies in the proposed indication to define benefits in both biomarker-positive and biomarker-negative cohorts depending on the specific prevalence of the biomarker in the intended population.

### Phenotype

PCWG4 recommends incorporating tumor phenotypic features into trials, including histology, protein expression, transcriptomic features, epigenetics, immunologic profile, and molecular imaging. Enhanced baseline and on-treatment phenotypic characterization should be considered with respect to the mechanism of action of the therapeutic agent and trial population. In addition, we recommend assessment for dominant resistance pathways including neuroendocrine differentiation and lineage plasticity when AR expression is low, or assays show discordant results.

## Response End Points

### Post-Treatment Prostate-Specific Antigen Declines/Nadir

Serum prostate-specific antigen (PSA) reflects a combination of tumor burden and differentiation status, and post-treatment PSA declines have been shown to be associated strongly with improvements in overall survival and quality of life across many disease states in advanced prostate cancer (Armstrong AJ, et al: Eur Urol Oncol 2:677-684, 2019; Halabi S, et al: J Clin Oncol 31:3944-3950, 2013; Armstrong AJ, et al: Eur Urol 86:552-562, 2024; Armstrong AJ, et al: J Clin Oncol 25:3965-3970, 2007; Small EJ, et al: Eur Urol Oncol 7:844-852, 2024; Saad F, et al: Eur Urol 86:329-339, 2024). PSA nadir after 6-12 months of initial induction hormonal or chemohormonal therapy is similarly strongly associated with prognosis and may identify subsets of patients with widely differing outcomes (Hussain M, et al: J Clin Oncol 24:3984-90, 2006; Harshman LC, et al: J Clin Oncol 36:376-382, 2018; Azad AA, et al: JAMA Netw Open 8:e258751, 2025). PCWG4 recommends reporting confirmed PSA declines and/or PSA nadir in a range of landmarks (3, 6, 12 months) as an intermediate end point in therapeutic trials, particularly for those agents that affect AR signaling or have cytotoxic properties. However, post-treatment PSA change lacks surrogate properties^67^ (Armstrong AJ, et al: J Clin Oncol 25:3965-3970, 2007; Gharzai LA, et al: Lancet Oncol 22:402-410, 2021; Gharzai LA, et al: NEJM Evid 2:EVIDoa2200195, 2023) and is not suitable alone as a regulatory end point.

### Pathologic Response in Neoadjuvant Therapy for Localized Prostate Cancer

Long median times to conventional outcomes are a barrier to developing new therapies in localized disease (McKay RR, et al: Cancer 130:1629-1641, 2024; McKay RR, et al: J Urol 205:1689-1697, 2021; McKay RR, et al: J Clin Oncol 37:923-931, 2019; McKay RR, et al: Prostate Cancer Prostatic Dis 21:364-372, 2018; McKay RR, et al: J Urol 206:80-87, 2021). Therefore, pathologic response assessment after neoadjuvant therapy is potentially analogous to radiographic response for advanced disease. In the context of PC neoadjuvant trials, prostatectomy pathologic response is prognostic but has not yet been validated as a surrogate marker. Nonetheless, pathologic complete response rates and minimal residual disease (5 mm of cancer or less) or percent residual viable tumor should be recorded as an outcome measure and may be useful for early phase trials to screen for efficacy depending on the mechanism of action.

### Circulating Tumor Cells

Circulating tumor cell (CTC) decline from baseline has been associated with survival in randomized controlled trials of various agents in patients with metastatic APMR prostate cancer. Both CTC conversion (a decline of CTC count from ≥5 CTC/7.5 mL of blood at baseline to <5 at 13 weeks, using an FDA-cleared assay, and CTC0 (a decline of CTC count from >0 at baseline to 0 at 13 weeks) are associated with survival (Heller G, et al: J Clin Oncol 36:572-580, 2018). It is not yet clear that CTC conversion and zero detectable CTCs (CTC0) are surrogate end points for survival in other disease states, including hormone therapy naïve disease, or with all drug classes, and confirmation of CTC decline is recommended to ensure durability of results. In addition, CTC detection rate at baseline is dependent on disease burden. These end points and changes in CTCs over time could be helpful, in conjunction with other outcomes, for increasing confidence in a novel therapeutic agent.

### Circulating Tumor DNA

Similar to CTCs, a decline in ctDNA content or tumor fraction (TF) from baseline has been associated with survival and progression end points in metastatic prostate cancer (Tolmeijer SH, et al: Clin Cancer Res 29:2835-2844, 2023; Conteduca V, et al: Br J Cancer 123:982-987, 2020). However, the supporting data for ctDNA in prostate cancer are limited at this time and significant validation is required (Wyatt AW, et al: Clin Cancer Res 30:5034-5041, 2024). Though also dependent on disease burden and cell-free DNA content, ctDNA may have a higher sensitivity of detection at baseline compared with CTCs, but various assays and definitions of ctDNA amount and decline have been proposed. ctDNA and ctDNA TF testing (Sweeney CJ, et al: Clin Cancer Res 30:4115-4122, 2024) at baseline and decline at 4 and 12 weeks should be explored in randomized trials, and the assay, analytic validation, and quantification methods should be clearly defined. Confirmation of ctDNA decline is recommended to ensure durability of results.

## Delay/Prevent End Points

### Patient-Centered End Points

Patient-centered end points include time to quality-of-life (QOL) deterioration (Morgans AK, et al: J Clin Oncol 36:1088-1095, 2018; Rush HL, et al: J Clin Oncol 40:825-836, 2022), time to symptomatic deterioration (Fizazi K, et al: Lancet Oncol 21:1513-1525, 2020), and time to pain progression (Basch EM, et al: Eur Urol 75:929-937, 2019). Disease-related symptoms should be reported distinctly from treatment-related symptoms (FDA: https://www.fda.gov/media/149994/download, 2024; Amdal CD, et al: Lancet Oncol 26:e683-e693, 2025). These end points should ideally use patient-reported outcome (PRO) assessments in addition to or instead of traditional physician assessments (Morgans AK, et al: Eur Urol 68:891-898, 2015; Agarwal N, et al: J Urol 206:914-923, 2021). We propose that these be incorporated similar to those outlined in the Response section as dual primary end points or as part of the family of primary end points, rather than as sole primary end points, to estimate overall benefit-risk ratios of novel therapies.

### Freedom From Subsequent Therapy End Points

Freedom from subsequent therapy end points may include time to next systemic therapy and eugonadal (testosterone >150 ng/dL) progression-free survival for those on previous androgen deprivation therapy and not on androgen receptor pathway inhibitor therapy (Tang C et al: JAMA Oncol 9:825-834, 2023; Shiota M, et al: Curr Med Res Opin 38:1351-1359, 2022; Smith MR, et al: Clin Cancer Res 27:4539-4548, 2021). The value of time to next systemic therapy as an end point must weigh the risks of the current therapy under study against the risks/benefits of the subsequent therapy and is of lower value if both therapies have similar toxicity. PCWG4 recommends defining PFS2 as the time interval from initial random assignment to the second radiographic or clinical progression after subsequent therapy or crossover although the time from subsequent or crossover therapy to second progression should also be recorded. The value of PFS2 as an end point reflective of patient benefit is greatest if the crossover or subsequent therapy criteria are clearly articulated and crossover/subsequent therapy options are finite and predefined, there is limited and uninformative censoring, and if the timing of subsequent assessments are pre-defined and standardized (Denmeade SR, et al: J Clin Oncol 39:1371-1382, 2021). PFS2 may be more relevant in earlier disease state settings. Inclusion of crossover designs also addresses the optimal timing of novel interventions.

### General Laboratory-Based Analytes

Alkaline phosphatase, bone-specific alkaline phosphatase, carcinoembryonic antigen, chromogranin-A, prosatic acid phosphatase, and lactate dehydrogenase (LDH) are examples of blood-based analytes that have shown prognostic value and may help to determine whether a patient is benefiting from treatment. For example, a rising alkaline phosphatase may indicate disease progression, even if bone imaging is equivocal. However, it is critical to note that these analytes often fluctuate, and some are relatively nonspecific (eg, LDH). Capturing the relationship between these changes over time and clinical symptoms and/or imaging-based and survival is also suggested. Changes in molecular testing (biopsies, ctDNA) results over time should also be recorded but are not recommended as an end point or reason to change therapy without prospective validation.

**TABLE A1.** Mapping of Previous Disease State Terms to New PCWG4 Disease State Terminology

PCWG3 Terminology	PCWG4 Terminology
Localized	Localized
Rising PSA noncastrate^a^	Nonmetastatic APMN if no previous APM APMS if responded to previous APM
nmCRPC	Nonmetastatic APMR Metastatic (PET only) APMR
Clinical metastases Noncastrate^b^	Metastatic APMN if no previous APM APMS if responded to previous APM
mCRPC Specify lines of therapy	Metastatic APMR Specify previous therapies

NOTE. In each disease state, PCWG4 recommends reporting the specific imaging modality used to define the state (CT, MRI, bone scan, PET).

Abbreviations: APM, androgen pathway modulation; APMN/S/R, androgen pathway modulator–naïve/sensitive/resistance; BCR, biochemical recurrence; CT, computed tomography; mCSPC, metastatic castration-sensitive prostate cancer; mHSPC, metastatic hormone-sensitive prostate cancer; MRI, magnetic resonance imaging; PCWG4, Prostate Cancer Working Group 4; PET, positron emission tomography; PSA, prostate-specific antigen.

^a^ Also known as BCR.

^b^ Also known as mHSPC or mCSPC.

**TABLE A2.** Suggested Validated Patient-Reported Outcomes for Inclusion in Specific Contexts of Use for Men With Prostate Cancer

Domain	Suggested Patient-Reported Outcome Measures
Prostate cancer–specific quality of life	EORTC QLQ-PR25 (prostate)^1^ EPIC—Full length^2^ FACT-P^3^ Targeted Radionuclide Therapy—FACT-RNT^4^
Overall health-related quality of life	EORTC QLQ-C30 (quality of life of patients with cancer)^5^ FACT-G (general)^6^ EQ-5D-5L^7^
Specific symptom measures	Any symptom NCI Patient Reported Outcomes version of the Common Terminology Criteria for Adverse Events (PRO-CTCAE) Measurement System^8^ PROMIS^9^ Prostate Cancer Symptom Group EPIC-26-short form^2^ EPIC-clinical practice^10^ FACT-RNT^11^ (*radionuclide-specific*) Pain Numeric Rating Scale^12^ Brief Pain Inventory (short form)^13^ FACT-BP (bone pain)^14^ Patient-reported analgesic use log^12^ PROMIS Cancer Bank v1.1—Pain Interference^15^ Fatigue Brief Fatigue Inventory^16^ EORTC QLQ-FA12 (cancer-related fatigue)^17^ FACIT-F^18^ PROMIS—Cancer Bank v1.0 Fatigue^19^ Neuropathy EORTC QLQ-CIPN20 (chemotherapy-induced peripheral neuropathy)^20^ FACT/GOG-NTx (neurotoxicity)^21^ FACT taxane^22^ PNQ^23^ Hormonal symptoms EPIC hormonal^24^ Hot flash frequency and hot flash score^25^ Hot flash–related daily interference^26^ Cognitive side effects FACT-Cog (cognitive function)^27^ PROMIS Bank v2.0—Cognitive Function^28^ Urinary symptoms EPIC urinary domain^29^ FAIT-U^30^ IPSS^31^ Sexual function EPIC Sexual Domain^32^ PROMIS Sex Function and Satisfaction v2.0 Full Profile Male^33^
Physical function	PROMIS—Cancer Bank v1.1—Physical Function^34^ SF-36 Physical Function subscale^35^ Physical activity metrics from wearable devices

NOTE. To minimize participant burden and provide the most meaningful data and relevance, only select assessments focused on expected outcomes and toxicity should be used.

Abbreviations: EORTC QLQ, European Organization for Research and Treatment of Cancer Quality of Life Questionnaire; EPIC, Expanded Prostate Cancer Index Composite; EQ-5D-5L, EuroQol 5-dimensions, 5-level; FACIT-F, Functional Assessment of Chronic Illness Therapy-Fatigue; FACT-P, Functional Assessment of Cancer Therapy-Prostate; FACT-RNT, Functional Assessment of Cancer Therapy-Radionuclide Therapy; FAIT-U, Functional Assessment of Incontinence Therapy-Urinary; IPSS, International Prostate Symptom Score; PNQ, Participant Neurotoxicity Questionnaire; PROMIS, Patient-Reported Outcomes Measurement Information System; SF-36, short form-36.

^1^ European Organisation for Research and Treatment of Cancer: EORTC QLQ—PR25. https://www.eortc.org/app/uploads/sites/2/2018/08/Specimen-PR25-English-1.1.pdf.

^2^ Expanded Prostate Cancer Index Composite (EPIC). https://medschool.umich.edu/departments/urology/research/quality-life-tools/epic.

^3^ Functional Assessment of Cancer Therapy-Prostate (FACT-P). https://www.facit.org/_files/ugd/626819_bcdd612dbf734297a32172aa2873d7f4.pdf.

^4^ Gudenkauf LM, Chavez MN, Maconi ML, et al: Developing a Patient-Reported Outcome Measure for Radionuclide Therapy for Prostate Cancer. J Nucl Med 64:869-872, 2023

^5^ EORTC QLQ-C30 (Quality of Life of Cancer Patients). https://www.eortc.org/app/uploads/sites/2/2018/08/Specimen-QLQ-C30-English.pdf.

^6^ FACT-G (General). https://www.facit.org/_files/ugd/626819_acb819ba51fd4552807feef38250db3f.pdf.

^7^ EuroQol 5-dimensions, 5-level (EQ-5D-5L). https://euroqol.org/.

^8^ NCI Patient Reported Outcomes version of the Common Terminology Criteria for Adverse Events (PRO-CTCAE) Measurement System. https://healthcaredelivery.cancer.gov/pro-ctcae/pro-ctcae_english.pdf

^9^ Patient-Reported Outcomes Measurement Information System (PROMIS). https://www.healthmeasures.net/.

^10^ Chang P, Szymanski KM, Dunn RL, et al: Expanded prostate cancer index composite for clinical practice: development and validation of a practical health related quality of life instrument for use in the routine clinical care of patients with prostate cancer. J Urol 186:865-72, 2011.

^11^ Functional Assessment of Cancer Therapy—Radionuclide Therapy (FACT-RNT). https://www.facit.org/measures/FACT-RNT.

^12^ Basch E, Trentacosti AM, Burke LB, et al: Pain palliation measurement in cancer clinical trials: The US Food and Drug Administration perspective. Cancer 120:761-767, 2014.

^13^ Brief Pain Inventory (Short Form). http://www.npcrc.org/files/news/briefpain_short.pdf

^14^ FACT-BP (Bone Pain). https://www.facit.org/_files/ugd/c0dc3a_2fd5b26f630b4d5990ea5c5743d893ee.pdf.

^15^ PROMIS Cancer Bank v1.1—Pain Interference. https://www.healthmeasures.net/.

^16^ Brief Fatigue Inventory. https://www.mdanderson.org/content/dam/mdanderson/documents/Departments-and-Divisions/Symptom-Research/BFI_English_SAMPLE.pdf.

^17^ EORTC QLQ-FA12 (Cancer-Related Fatigue). https://www.eortc.org/app/uploads/sites/2/2018/08/Specimen-FA12-English.pdf.

^18^ Functional Assessment of Chronic Illness Therapy-Fatigue (FACIT-F). https://www.facit.org/_files/ugd/626819_d1beda1cf9b14857879e082ac7682220.pdf.

^19^ PROMIS—Cancer Bank v1.0 Fatigue. https://www.healthmeasures.net/.

^20^ EORTC QLQ-CIPN20 (Chemotherapy-Induced Peripheral Neuropathy). https://www.eortc.org/app/uploads/sites/2/2018/08/Specimen-CIPN20-English.pdf.

^21^ FACT/GOG-NTx (Neurotoxicity). https://www.facit.org/_files/ugd/626819_bb41f4bd0c89499a8675a32e138e4ce2.pdf.

^22^ FACT taxane. https://www.facit.org/_files/ugd/626819_f902fdea55424b4aa7f5aa19a51ed08c.pdf.

^23^ Hausheer FH, Schilsky RL, Bain S, et al: Diagnosis, management, and evaluation of chemotherapy-induced peripheral neuropathy. Semin Oncol 33:15-49, 2006.

^24^ EPIC hormonal. https://medschool.umich.edu/departments/urology/research/quality-life-tools/epic.

^25^ Sloan JA, Loprinzi CL, Novotny PJ, et al: Methodologic lessons learned from hot flash studies. J Clin Oncol 19:4280-4290, 2001.

^26^ Carpenter JS: The Hot Flash Related Daily Interference Scale: a tool for assessing the impact of hot flashes on quality of life following breast cancer. J Pain Symptom Manage 22:979-989, 2001

^27^ FACT-Cog (Cognitive Function). https://www.facit.org/_files/ugd/626819_2f2007bdd64643539d44da7da436f8e2.pdf.

^28^ PROMIS Bank v2.0—Cognitive Function. https://www.healthmeasures.net/.

^29^ EPIC Urinary Domain. https://medschool.umich.edu/departments/urology/research/quality-life-tools/epic.

^30^ Functional Assessment of Incontinence Therapy—Urinary (FAIT-U). https://www.facit.org/_files/ugd/626819_fa26317973ce466d8f262cecd7caa00c.pdf.

^31^ Lerner LB, McVary KT, Barry MJ, et al: Management of lower urinary tract symptoms attributed to benign prostatic hyperplasia: AUA GUIDELINE PART I-initial work-up and medical management. J Urol 206:806-817, 2021.

^32^ EPIC Sexual Domain. https://medschool.umich.edu/departments/urology/research/quality-life-tools/epic.

^33^ PROMIS Sex Function and Satisfaction v2.0 Full Profile Male. https://www.healthmeasures.net/.

^34^ PROMIS—Cancer Bank v1.1—Physical Function. https://www.healthmeasures.net/.

^35^ Ware JE, Jr., Gandek B: Overview of the SF-36 Health Survey and the International Quality of Life Assessment (IQOLA) Project. J Clin Epidemiol 51:903-912, 1998.

**TABLE A3.** Established Prognostic Factors of Overall Survival

Factor
Age
Race
Ethnic background
Social determinant of health
Area deprivation index
Hemoglobin
Performance status
PSA
N-telopeptide, pyridinoline, C-terminal collagen propeptide
Metastatic disease (at diagnosis, present stage)
Grade group and histology (small cell, double-negative, AR+)
Albumin
LDH
Volume of metastatic disease (CT, bone scan, MRI, PET)
Alkaline phosphatase
Site of metastases (visceral [liver/lung/adrenal], bone or lymph nodes [N1 or M1])
Neutrophil/lymphocyte ratio
Germline and somatic pathogenic variants
Pain
PSA doubling time
Type of progression
No. of previous treatments
Type of previous treatment
PSMA imaging (for PSMA targeted therapies) features or FDG PET features
Stage at diagnosis
Tumor genetic biomarkers: *TP53, CDK12, ATM, PTEN, RB1, BRCA1, BRCA2, SPOP, MYC, AR, AR-V7*, others Germline genetic biomarkers: Homologous recombination repair alterations such as *BRCA2, ATM; TP53, CHEK2,* *HSD3B1*, and others
Time from diagnosis
Circulating tumor cell enumeration
ctDNA tumor fraction

Abbreviations: AR, androgen receptor; CT, computed tomography; ctDNA, circulating tumor DNA; FDG, fluorodeoxyglucose; LDH, lactate dehydrogenase; MRI, magnetic resonance imaging; PET, positron emission tomography; PSA, prostate-specific antigen; PSMA, prostate-specific membrane antigen.
